# Demand‐Side Management for a Decentralised and Equitable Energy Transition in Aotearoa New Zealand

**DOI:** 10.1002/snz2.70006

**Published:** 2026-02-15

**Authors:** Baxter Kamana‐Williams, R. J. Hooper, Alice E. Cerdeira, J. Geoffrey Chase

**Affiliations:** ^1^ Department of Mechanical Engineering University of Canterbury Christchurch New Zealand; ^2^ Keough School of Global Affairs University of Notre Dame Notre Dame Indiana USA; ^3^ Maidstone Associates Ltd Christchurch New Zealand

**Keywords:** agent‐based model, decentralisation, demand‐side management, electricity transition, energy transition, just transition

## Abstract

Aotearoa New Zealand's shift towards a sustainable, decentralised electricity system will involve local generation, greater consumer empowerment and more active intervention at both local and grid levels. Demand‐side management (DSM) will play a key role in achieving these goals. However, the complexity of electricity demand and the risk of unintended consequences mean decentralisation must be approached with care, considering the interests of all electricity sector participants, including residential consumers from across the socioeconomic spectrum. This paper highlights key considerations for this transition, informed by recent, validated agent‐based modelling research targeted at residential consumers, which should inform policy: (i) behavioural dynamics, (ii) electricity pricing structures, (iii) socioeconomic variations in benefits of distributed generation and storage, (iv) the importance of energy efficiency and (v) consumer trust.

1

Summary Aotearoa New Zealand's shift towards a decentralised energy system, featuring greater local generation, greater consumer empowerment, and more resilient grid operations, requires smart, equitable, and behaviourally informed demand‐side measures. Residential demand‐side management (DSM), when carefully designed, is a critical “bottom‐up” enabler of this transition, but poor design risks increasing social inequality and burden. The reports supporting the conclusions presented here are referenced. This work distils these findings, highlighting important considerations for this Viewpoint on Aotearoa New Zealand's energy transition.

## 2 Introduction

Climate change and energy security concerns are driving changes in Aotearoa New Zealand's electricity system, including increased electrification (Transpower 2021) and renewable generation (Pimentel Pincelli et al. 2024). Decentralisation, shifting from large‐scale electricity generation to smaller‐scale distributed energy resources (DERs), is a key component of this transition (Brent et al. 2025), which can “*provide for more of our power needs at the local level*, *in ways that work for us*” (Electricity Authority 2025). However, these changes bring challenges, including increased electricity system costs (Tibi et al. 2022) and potential inequities in the distribution of costs and benefits (Wang and Lo 2021).

Demand‐side management (DSM) can address these challenges by increasing consumer participation, reducing costs and peak demand and increasing utilisation of intermittent renewable generation (Williams et al. 2023). Demand‐side interventions can deliver flexibility and resilience, often with better cost‐efficiency than supply‐side infrastructure (Maxim and Grubert 2023) and are a strong enabler of Aotearoa New Zealand's energy transition (Stephenson et al. 2018; Williams and Bishop 2024).

However, poor design, planning and implementation of DSM and decentralisation will introduce unintended consequences. First, flawed incentives can encourage undesirable emergent behaviour, such as increasing peak loads (Bishop et al. 2023). Second, demand‐side interventions can increase inequality and energy poverty (Henriksen et al. 2025), especially as programs are often designed for one type of (usually rich) consumer at the expense of others (Strengers 2014).

This viewpoint highlights considerations for DSM in Aotearoa New Zealand, drawn from our recent modelling and wider energy research. Our models are validated by comparing outputs with real electricity demand from residential low‐voltage transformers across seasons, locations and demographics, with correlation between modelled and real outputs above 0.8 in over 80% of cases (Williams, Hooper, et al. 2025). Sensitivity analyses also show the importance of accurate behavioural inputs, affirm the validity of these models for behavioural analyses and demonstrate the value of agent‐based modelling for incorporating consumer needs and outcomes (Kamana‐Williams et al. 2025). This modelling approach can strengthen the assumptions underpinning energy policy and regulatory design, and help shape a clearer, evidence‐based vision of Aotearoa New Zealand's future electricity system.

**FIGURE 1:**
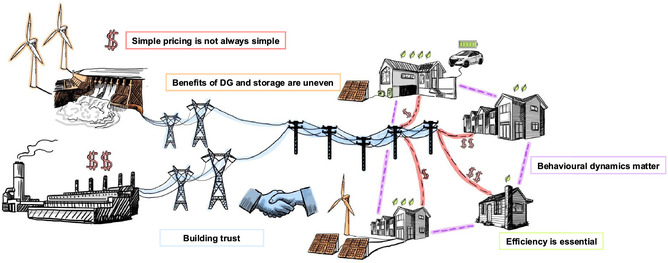
Considerations for a decentralised and empowered residential energy future in Aotearoa New Zealand.

Original data, methodologies and validation are presented in our previous publications (Kamana‐Williams et al. 2025; Williams, Hooper, et al. 2025). Key findings are summarised under five interconnected themes, illustrated in Figure 1 and detailed in Section 2.

## 3 Key Findings

### 3.1 Behavioural Dynamics Matter

DSM program design must reflect real household behaviours to deliver reliable and equitable outcomes. These behaviours, and their intra‐ and inter‐personal variability, can be very accurately captured with simple dynamic models, such as agent‐based models (ABMs) (Kamana‐Williams et al. 2024). ABMs are “bottom‐up” models of individuals (“agents”), in which aggregate group behaviours arise from the sum of agents’ actions and interactions. ABMs are well‐suited to modelling situations in which both individual and aggregate behaviours are important (Shinde and Amelin 2019), such as the residential energy sector. These models accurately integrate behavioural data and can consider outcomes beyond just electricity system considerations, including equity, consumer comfort and emissions reductions, so they can better support DSM program design through accurate predictions of uptake and human outcomes.

### 3.2 Simple Pricing Is Not Always Simple

Time‐of‐use tariffs can reduce household costs and peak demand (Carmichael et al. 2021), but risk backfiring and increasing peak demand without smart device uptake or consumer support (Kamana‐Williams et al. 2025), such as by unintentionally incentivising households to shift consumption to the same off‐peak time, creating secondary peaks. Alternative electricity pricing structures, such as maximum demand tariffs (Subramani et al. 2017), can be designed to benefit consumers without increasing network stress, have been shown to be achievable in other countries (Liu et al. 2023; Hao et al. 2024) and should be implemented in Aotearoa New Zealand. However, any such programs must be fine‐tuned to avoid unintended consequences, highlighting a clear need for consumer‐centric program design coupled with smart, DSM‐ready technologies to support flexible and equitable participation.

### 3.3 Benefits of Distributed Generation and Storage Are Uneven

Distributed solar generation and battery storage can reduce peak demand, emissions and consumer costs (Iweh et al. 2021), and increase energy security and resilience (Williams, Croft, et al. 2025). However, high capital costs for installation mean uptake is typically low in low‐income households (Mathew and Nagaraja Pandian 2024), and financial benefits skew towards higher‐income households without targeted subsidies (Kamana‐Williams et al. 2025). Thus, policies must balance peak demand reduction and consumer affordability. Without subsidies or programs supporting equitable access, DERs could deepen energy inequality.

### 3.4 Efficiency Is Essential

Increasing energy efficiency reduces peak demand and offers public health and equity benefits, especially when targeted to low‐income communities. Highest net savings arise from boring but expected solutions, such as installing (additional) building insulation and replacing inefficient space heaters with heat pumps (Kamana‐Williams et al. 2025), driven by the measured health system savings of warmer, drier homes (Grimes et al. 2012). Energy efficiency should thus be the cornerstone of decentralisation policy, supporting grid benefits, public health and equity.

### 3.5 Building Trust

Outcome variations across the socioeconomic spectrum highlight the importance of targeting decision‐making and policy interventions to maximise whole‐system outcomes, which can benefit a wider range of energy‐ and non‐energy‐system stakeholders. Mean values often inaccurately represent outcomes for the majority of households (Kamana‐Williams, Hooper, Steidl, et al. 2025), so, where possible, the full distribution of outcomes should be used to transparently inform energy policy. Interventions and subsidies should be designed considering energy equity and socioeconomic variations to ensure benefits are maximised and equally distributed. Thus, policy and regulatory interventions should be targeted to maximise both peak reduction, public health and social good.

## 4 Conclusions and Policy Implications

DSM is a key to Aotearoa New Zealand's energy transition. Policymakers should treat DSM not just as a technical solution, but as a strategic investment to bridge emissions reductions, energy equity and grid resilience. Accurate behavioural models and data should be used to inform program design, considering whole‐system outcomes and ensuring an equitable, sustainable energy future. Specific policy recommendations are as follows:

• Design programs supporting DSM and decentralisation with validated models reflecting real consumer behaviours and their variations across the socioeconomic spectrum.
• Pair dynamic electricity pricing with technology incentives and consumer education.
• Subsidise access to distributed solar generation and battery storage for low‐income households to ensure inclusive decarbonisation and peak demand reduction.
• Prioritise energy efficiency standards and grants for low‐income households as foundational tools for system resilience and social wellbeing.
• Design policies and regulations to build consumer trust and align with climate and equity goals, using public health outcomes, transparency and the distribution of financial benefits as key success indicators.

## Funding

The authors have nothing to report.

## Conflicts of Interest

The authors declare no conflicts of interest.
